# Digital Orthopedics: The Future Developments of Orthopedic Surgery

**DOI:** 10.3390/jpm13020292

**Published:** 2023-02-06

**Authors:** Zhonghai Li

**Affiliations:** 1 Department of Orthopedics, First Affiliated Hospital of Dalian Medical University, Dalian 116011, China; lizhonghaispine@126.com; Tel.: +86-18098876419; Fax: +86-411-83635963; 2 Key Laboratory of Molecular Mechanism for Repair and Remodeling of Orthopedic Diseases, Dalian 116000, China

Digital medicine is a new type of medical treatment that applies modern digital information technologies to entire medical procedures [1,2]. Digital technologies have played a vital role in the development of medical care, improving our ability to accurately diagnose and treat diseases and enhancing medical care services for individuals. As a new medical method that applies modern digital information technologies to the entire disease treatment process, digital medicine has attracted increasing attention in recent years [3,4]. Breakthroughs in digital technologies have gradually made digital healthcare relevant to an increasing number of clinical indications and application scenarios. For example, breakthroughs in cloud and Internet technologies have facilitated the empowerment of grassroots informatization; AI imaging and digital therapy have been used to provide an increasing number of indications; and the development of the Internet of Things and 5G technologies has made out-of-hospital health management a reality that could only have been imagined just a few years ago [5,6,7]. As various digital technologies continue to be integrated, unprecedented progress is being made in digital medical care.

Orthopedic digital medicine is an interdisciplinary subject based on orthopedics and assisted by computer image processing techniques. It involves fields such as biomechanics, human anatomy, materials science, mechanical engineering, solid geometry, electronics, and informatics. The primary applications of digital orthopedic technology include clinical computer-aided design/manufacturing, mechanical simulation of the human musculoskeletal system, 3D virtual simulation and visualization, finite element technology, surgical navigation and robot-assisted technology, and medical image processing and 3D modeling [8,9,10,11]. The application of various digital technologies can greatly improve the efficiency, accessibility, and capabilities of medical services and prompt active and timely intervention by physicians. In addition, orthopedic digital medical care can provide data-driven, personalized diagnosis and treatment and provide physicians with auxiliary diagnosis functions based on medical principles and data analysis models, thus prompting more efficient and effective diagnosis and treatment [12,13,14]. The medical system has historically emphasized treatment over the prevention of disease, while poor treatment compliance often results in less than satisfactory outcomes, let alone personalized precision treatment. In orthopedic digital medicine, digital technologies can be used to facilitate continuous monitoring of a patient’s musculoskeletal system to promote the development of good living habits, while also providing both the physician and patient with early warning indications, thus shifting the focus to disease prevention and subsequently more effective medical treatment. Furthermore, data-driven digital medical care can effectively provide a patient with personalized treatment plans based on the collection and analysis of the patient’s data. The technical composition and applications of digital orthopedics are shown in Figure 1.

Diseases of the musculoskeletal system have always had high morbidity and disability rates, and further research into their pathogenesis, diagnosis, and treatment methods is required. As orthopedics continues to evolve, personalization, precision, and minimal invasiveness should be prioritized. Making the diagnosis and treatment of musculoskeletal diseases safer and more convenient and improving the level of diagnosis and treatment can greatly benefit patients with musculoskeletal diseases. Research into orthopedic digital medicine and the associated clinical transformation is still in a period of rapid development. It is believed that with continuous development and innovation, orthopedic digital medicine will eventually form its own complete clinical system and disciplinary theory and will play a developmental role in the field of orthopedics as a whole.

## Figures and Tables

**Figure 1:**
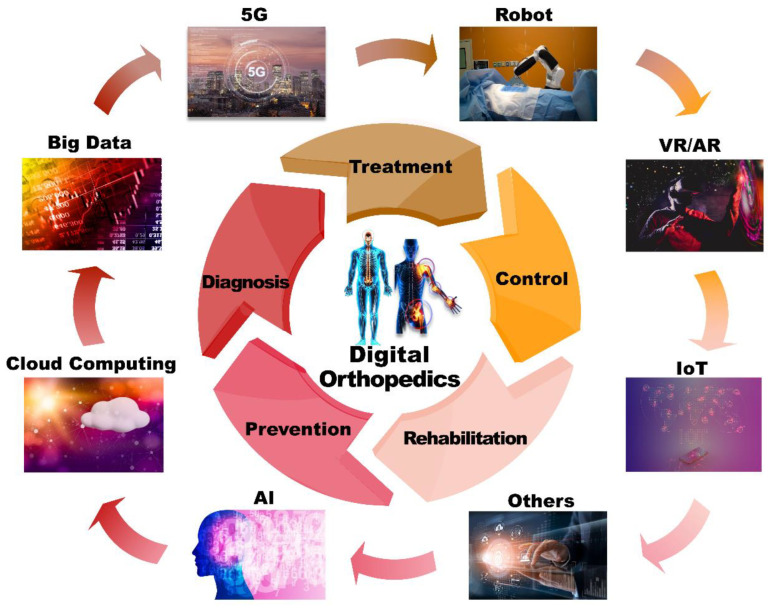
The technical composition and applications of digital orthopedics.

## Data Availability

Not applicable.
